# Expression of transcription factors and crystallin proteins during rat lens regeneration

**Published:** 2010-03-03

**Authors:** Yusen Huang, Lixin Xie

**Affiliations:** State Key Lab Cultivation Base, Shandong Provincial Key Lab of Ophthalmology, Shandong Eye Institute, Qingdao, P.R. China

## Abstract

**Purpose:**

To establish a model of lens regeneration in rats and to detect the expression of transcription factor and crystallin genes.

**Methods:**

An extracapsular lens extraction (ECLE) was performed in Sprague-Dawley rats. Examinations with slit-lamp and histological analysis were performed at various time points after ECLE. Real-time PCR and/or immunofluorescence were performed to detect the expression of the lens transcription factors paired box 6 (*Pax6*), prospero homeobox 1 (*Prox1*), and forkhead box E3 (*Foxe3*) and α-, β-, and γ-crystallin (*Cryaa*, *Cryab*, *Crybb1*, *Crybb2*, *Cryba2*, and *Crygd*, respectively).

**Results:**

Lens epithelial cells (LECs) were left behind under the anterior capsule immediately after ECLE. Lens fiber differentiation had occurred in the peripheral capsular bag in all rats 3 days after ECLE. One month after surgery, all capsular bags were filled with new semitransparent lenticular structures displaying an established equator with well differentiated bow regions. The mRNA-expression quantity of lens transcription factors and α-, β-, and γ- crystallin increased after ECLE. *Pax6* was expressed in both LECs and the newly regenerated lens fiber cells, *Prox1* was expressed both in LECs and differentiating lens fiber cells, and *Foxe3* was confined to LECs.

**Conclusions:**

Lens fiber differentiation during regeneration follows a process similar to embryological development, with proliferation of epithelial cells along the anterior and posterior capsule, elongation of the posterior epithelial cells, and differentiation of epithelial cells into lens fibers. The regenerated lens contains proteins and transcription factors similar to those found in normal lenses. Inductive interactions seen during lens development are not necessary for lens regeneration.

## Introduction

The ocular lens regenerates through two very different processes. In amphibian models, following the total removal of the lens and its capsule, lens regeneration is achieved by transdifferentiation of pigmented epithelial cells from the dorsal iris, as in newts, or of endothelial cells of the larval cornea, as in *Xenopus laevis*. Lens regeneration in mammals has been studied extensively in rabbits [1], and recently such studies have been extended to mice [2,3]. The source of the regenerated lens in these cases is adherent lens epithelial cells (LECs) that cannot be completely removed.

Vertebrate lens development initiates when a portion of lens-competent cephalic ectoderm responds to an inductive signal from the optic neuroepithelium. The induced ectoderm, known as the lens placode, thickens, invaginates, and pinches off to form the lens vesicle. Cells in the anterior portion of the lens vesicle develop into lens epithelium, whereas cells in the posterior portion leave the cell cycle, elongate, and differentiate into primary lens fibers. After the lens forms, its growth is mediated by lens epithelial proliferation and differentiation of LECs at the lens equator into secondary fiber cells, and α-, β-, and γ-crystallin proteins are then expressed in sequence [4,5]. These LECs remain proliferative during the rest of life, and the lens continues its growth by adding new layers of secondary lens fibers in its equatorial region. This differentiation event is marked by dramatic changes in cell shape and the accumulation of crystallin proteins [6].

Recently, molecular genetic techniques have been applied to the analysis of lens development. A series of transcription factors involved in early lens development have been identified, including paired box 6 (*Pax6*), prospero homeobox 1 (*Prox1*), forkhead box E3 (*Foxe3*), sex determining region Y-box 2 (*Sox2*), and SIX homeobox 3 (*Six3*), among others [7]. These transcription factors are critical for normal lens morphogenesis and the regulation of lens fiber differentiation.

Lens regeneration is a potential approach to restoring normal vision after cataract surgery. We used rats as an animal model for addressing the mechanism of len regeneration, using the frontline technologies of molecular biology.

## Methods

### Animals, surgeries, and tissue processing

A total of 48 2-month-old Sprague-Dawley rats (Beijing Laboratory Animal Research Center, Beijing, China), weighing 200–250 g were used in this study. Procedures adhered to the guidelines of the Association for Research in Vision and Ophthalmology Resolution on the Use of Animals in Ophthalmic and Vision Research. The animals were placed under general anesthesia with intravenous ketamine hydrochloride (60 mg/kg; Harvest Pharmaceutical, Shanghai, China) and xylazine (5 mg/kg; Pharma Chemical Plant, Nanjing, China), and topically anesthetized with Alcaine (Alcon Laboratories, Fort Worth, TX), after which their pupils were dilated with Mydrin-P (Santen Pharmaceutical, Japan). This surgical technique has been described previously [2]. In brief, a superior corneal incision was made with a 1 mm keratome, and a viscoelastic material (hyaluronic acid; Qisheng Biologic Preparation, Shanghai, China) was injected into the anterior chamber. Continuous circular capsulorhexis of the anterior capsule was performed using capsulorhexis forceps. The corneal incision was then extended to approximately 90° with Vannas scissors; this was followed by hydrodissection and lens removal. The incision was closed with continuous 11–0 nylon sutures. Following the procedure, topical Tobradex ointment (tobramycin 0.3% and dexamethasone 0.1%; Alcon Laboratories) and atropine ointment were administered.

The rats (n=6 per time point) were killed with an overdose of ketamine hydrochloride at 0 h, 1, 3, 7, 14 days, 1, 2, and 3 months after surgery. Before death, they were anesthetized, and the anterior segment or eyeball was evaluated by operating microscopy. The status of the cornea, posterior capsule, pupil, and iris was recorded.

### Histological analysis

Samples for routine histology were collected in cold phosphate-buffered saline (PBS) and fixed in 10% neutral buffered formalin for 24 h. They were frozen in optimal cutting temperature optimal cutting temperature (OCT) compound, cut into 10~14 µm sections, and stained with hematoxylin and eosin.

Lens tissue collected at 3, 7, and 14 days as well as 1 and 3 months after surgery were subjected to immunofluorescence using 12~16 µm cryosections. The cryosections were placed in 1:1 acetone: methanol pre-chilled to −20 °C and incubated for 10 min or longer at −20 °C.The sections were blocked for 1 h with blocking solution (10% normal serum in PBS), incubated for 2 h with primary antibodies, washed with PBS, incubated for 40 min with secondary antibodies, washed with PBS again and mounted with UltraCruz^TM^ mounting medium (Santa Cruz Biotechnology, Santa Cruz, CA). All incubations were performed at 37 °C. Primary antibodies used were polyclonal anti-Pax6 (R&D Systems, Minneapolis, MN) at a 1:300 dilution, polyclonal anti-Prox1 (Abcam, London, UK) at a 1:300 dilution, and polyclonal anti-Foxe3 (Abcam) at a 1:50 dilution. Secondary antibodies were goat anti-mouse IgG, goat anti-rabbit IgG, and rabbit anti-goat (Tianjin Haoyang Biologic Manufacture, Tianjin, China) at a 1:400 dilution. The sections were counterstained with DAPI to visualize nuclei. For negative controls, secondary antibodies alone were used, without a primary antibody treatment.

Images for all histological analyses were captured using a Nikon E800 (Nikon Instruments, Tokyo, Japan) microscope and a Nikon DS-U1 digital camera. The green staining corresponds to Pax6, Prox1 and Foxe3 immunolabeling, while the blue staining corresponds to cell nuclei.

Lens tissue collected at 14 days and 1, 2 and 3 months after surgery (regenerated lens) and 2 month after birth (normal lens) were weighed. After being washed with normal saline and placed on filter papers to absorb the saline for 30s, the lens tissues were weighed using an electron balance.

### RNA extraction and cDNA preparation

Lens tissues were collected before surgery, immediately after extracapsular lens extraction (ECLE), and 3 days, 14 days, 1 month and 3 months after surgery. The total RNA from the samples collected at different time points was extracted using the EZ-10 spin column RNA purification kit (Bio Basic, Toronto, Canada), and RNA concentrations were determined. PCR reactions (total volume of 20 ml) were performed using an AMV first-strand cDNA synthesis kit (Bio Basic) according to the manufacturer’s instructions. The cDNA was used to detect the quantity of the transcription factors of *Pax6* and *Prox1* and crystallin genes; *Cryaa*, *Cryab*, *Crybb1*, *Crybb2*, *Cryba2*, and *Crygd* (which encode αA-, αB-, βB1-, βB2-, βA2-, and γD-crystallin, respectively) by RT–PCR and quantitative real-time PCR.

### RT–PCR and sequencing analysis

Primer pairs for PCR amplification of target genes are shown in Table 1. PCR conditions were 94 °C for 2 min, 94 °C for 45 s, followed by annealing and extension at 72 °C for 40 s each. After amplification, the samples were run on a 2% agarose gel and analyzed for bands of the expected sizes. All primers were optimized for annealing temperatures and cycles. PCR products were then run on a 1% agarose gel, the DNA fragments were cut from the gel with a clean scalpel, and DNA for sequencing was extracted. Sequencing of the samples was done using the ABI PRISM 377 DNA Sequencer (Applied Biosystems, Foster City, CA) using standard manufacturer protocols (Big Dye Terminator kit; Applied Biosystems). The sequences obtained were compared with the corresponding normal gene sequences.

**Table 1:** Primers for RT–PCR.

**Gene name**	**Primers (5′–3′)**	**Size of product (bp)**	**Annealing temperature (°C)**	**Cycles**
*Pax6*	F: 5′GACAGGGAGAAAACACCAACTC 3′	336	62.9	28
	R: 5′ TGGTACTGAAACTGCTGCTGAT 3′			
*Prox1*	F: 5′TGTTCTTTTACACCCGTTACCC 3′	363	62.1	35
	R: 5′CACTATCCAGCTTGCAGATGAC 3′			
*Cryaa*	F: 5′GAGGGCTGAGGATTTGAGA 3′	268	53.1	26
	R: 5′ ATACGCCTGCGGTAAGTG 3′			
*Cryab*	F:5′ GGCTAACCGACTCTACACTCA 3′	483	58.5	26
	R:5′ CTGTTTCCTTGGTCCATTCA 3′			
*Crybb1*	F: 5′ ACGATGGGACACCTGGAC 3′	206	57.9	26
	R: 5′ CACTGGAGACGGTTATACTGC 3′			
*Crybb2*	F: 5′ CCTCAGACCACCAGACACAG 3′	364	61.3	26
	R: 5′ CTCCATCTTCTTGCCCGTAA 3′			
*Cryba2*	F: 5′ CTCTGGGACGAGGAGGACTT 3′	352	61.7	26
	R: 5′ ATGGAAGGCAGTGATGGGTA 3′			
*Crygd*	F: 5′ AGCACAGACCACTCCAACCT 3′	330	62.0	26
	R: 5′ AGCCCTCCAGCACATTGA 3′			
*G3pdh*	F: 5′ACCACAGTCCATGCCATCAC 3′	439	60.7	24
	R: 5′TCCACCACCCTG TTGCTGTA 3′			

In the “Primers” column F indicates forward primer and R indicates reverse primer.

### Quantitative PCR conditions

Real-time PCRs were performed with the TaqMan probe method for *Pax6* and *Prox1* and with SYBR I Green chemistry for crystallin genes. Primers used for amplification of target genes are shown in Table 2. The TaqMan probes were designed using Primer Express Software (ABI). For the TaqMan probe method, the probe was labeled at the 5′ end with the reporter dye FAM and at the 3′ end with the quencher dye TAMRA. The primers used for amplification of the crystallin genes were the same as those used for RT–PCR.

**Table 2:** Primers for real-time PCR.

**Method**	**Gene name**	**Primer (5’–3’)**
TaqMan probe	*Pax6*	F: 5’-CGTGCGACATTTCCCGAATT-3’
		R: 5’- TCTTGGCTTACTGCCTCCGAT-3’
	*Pax6* TaqMan probe	5’-TGCAGGTGTCCAACGGATGTGTGA-3’
	*Prox1*	F: 5’-AGTGAAAAGGACGGTAGGGA-3’
		R: 5’- GCGTGTTGCACCACAGAAT-3’
	*Prox1* TaqMan probe	5’-TGCTAAGGCAAGGGCAACATTTT-3’
	*Gapdh*	F: 5’-TGGAGTCTACTGGCGTCTT-3’
		R: 5’-TGTCATATTTCTCGTGGTTCA-3’
	*Gapdh* TaqMan probe	5’-CTGAAGGGTGGGGCCAAAAG-3’
SYBR I Green chemistry	*Gapdh*	F: 5’-CCCATCTATGAGGGTTACGC-3’
		R: 5’- TTTAATGTCACGCACGATTTC-3’

In the “Primer” column, F indicates forward primer and R indicates reverse primer.

Real-time PCR was performed using the FTC2000 (Funglyn Biotech, Toronto, Ontario, Canada). Samples were set up in 50 µl final volumes containing 6 µl 5× PCR buffer, 0.6 µl 2× primers (25 pmol/µl), 0.3 µl probe (25 pmol/µl) or 0.3 µl SYBR I Green, 1 µl dNTPs (10 mM), 0.3 µl Taq enzyme (5 U/µl), 3 µl Mg^2+^ (25 mM), 1 µl template and 17.2 µl DEPC water (Sigma). Amplification conditions consisted of 4 min at 94 °C, followed by forty 20-s cycles at 94 °C and finally 30 s at 60 °C. The relative expression was calculated based on the expression of glyceraldehyde-3-phosphate dehydrogenase (*Gapdh*).

## Results

### Clinical observations

**Figure 1:**
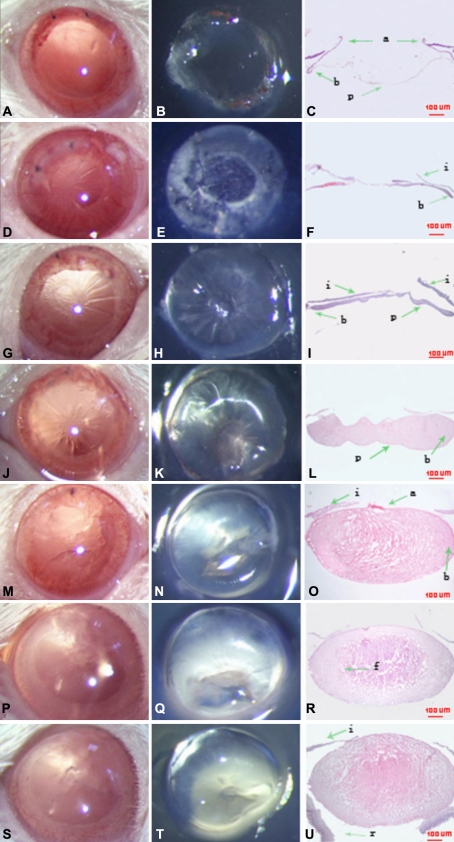
Morphological examination of rat lens regeneration.  Photographs of a rat eye (**A**, **D**, **G**, **J**, **M**, **P**, **S**), lens tissue (**B**, **E**, **H, K**, **N**, **Q**, **T**), and histologic analysis (**C**, **F**, **I**, **L**, **O**, **R**, **U**; hematoxylin and eosin staining) after ECLE. Continuous circular capsulorhexis of the anterior capsule and clear posterior capsule in a rat eye immediately after ECLE (**A**, **B**, **C**). On day 3, PCO is noted (**D**, **E**, **F**). On day 7, the eye shows development of clinically evident PCO (**G**, **H**), and Seomerring’s ring forms (**G**, **H**, **I**). On day 14, elongated lens fibers on the posterior capsule become well differentiated lens fibers (**J**, **K**, **L**).One month after ECLE, the region of anterior capsulorhexis is opaque, and the capsular bag is full of regenerated semitransparent lens material (**M**, **N**, **O**). Two to three months after ECLE (**P-U**), the regenerated lens is almost similar in size to the intact. Note the relatively loose packing of the elongating fiber cells. Abbreviations: a, anterior capsule; b, bow regions; c, cornea; e, lens epithelium; f, lens fiber cells; i, iris; p, posterior capsule; r, retina.

**Figure 2:**
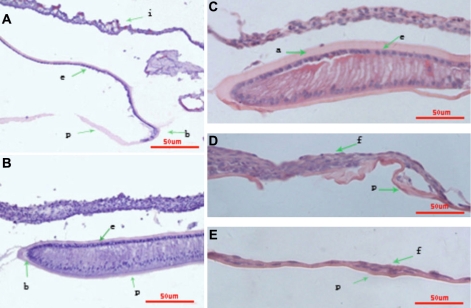
Hematoxylin and eosin staining of lens tissue after ECLE. The capsular bag is open, and residual LECs are observed on the peripheral anterior capsule immediately after ECLE (**A**). On day 3, LECs covered the inner surface of the anterior and posterior capsules, and LECs lining the posterior capsule show early changes characteristic of lens fiber differentiation (**C**). The LECs are multilayered (**D**) and migrate to the central posterior capsule. Some cells are spindle-shaped (**E**). On day 7, some cells are spindle-shaped. The nuclei of LECs lining the posterior capsule migrated away from the basement membrane (**B**).

The postoperative statuses are shown in Figure 1 and Figure 2. Posterior capsule folds were noted at 3 days after ECLE (Figure 1D,E). All eyes exhibited clinically evident second cataracts and Seomerring’s rings at 1 week (Figure 1G,H). Two weeks after surgery, Seomerring’s rings were more significant (Figure 1J,K). All capsular bags were filled with new semitransparent lenticular structures at 1 month, which continued to increase at a slow pace during the following 1 to 2 months.

The eyes appeared well healed, with clear corneas and deep anterior chambers, in 39 rats (81.3%). Two eyes had corneal opacification, and one eye had large amounts of fibrin in the anterior chamber caused by hyphema. Posterior synechiae were present in six eyes.

The average wet weights of regenerated lenses were 0.0063±0.0005 g, 0.0130±0.0016 g, 0.0198±0.0006 g, and 0.0223±0.0014 g at 14 days, 1, 2, and 3 months after surgery, respectively. The wet weights of the normal lenses were 0.0261±0.0005 g. When compared to the normal lenses, the average wet weight of the regenerated lenses showed a statistically significant increase (ANOVA, p=0.000).

### Lens fiber differentiation

Some residual LECs were observed on the peripheral anterior capsule immediately after surgery. Scarce residual lens matter was present inside the capsular bag. The anterior capsule at the capsulotomy margin rolled upwards, and the capsular bag was open. No contact between the anterior and posterior lens capsule occurred (Figure 1C and Figure 2A).

On the first day after surgery, the rolling-up anterior capsule at the capsulotomy margin adhered to the posterior capsule and LECs had migrated toward the center of the capsular bag. On the third day, a monolayer of LECs covered the inner surface of the anterior and posterior capsules in the capsular bag (Figure 1F and Figure 2C). LECs lining the posterior capsule appeared to show early changes characteristic of lens fiber differentiation. The LECs at the adhesive portion were multilayered and migrated to the central posterior capsule from the adhesive portion, and some cells were spindle-shaped (Figure 2D,E). On the seventh day, new lens fibers continued to increase. The capsular bag was filled with fibers and formed the Soemerring's ring (Figure 1I), the nuclei of LECs lining the posterior capsule migrated away from the basement membrane (Figure 2B), and vacuolation was present in the new lens fibers. On the 14th day, the lens fibers on the posterior capsule were found to have elongated extensively and become well differentiated lens fibers (Figure 1L). One month after lensectomy, the capsule was full of differentiated lens fibers, displaying an established equator with well differentiated bow regions (Figure 1O). At the end of the experiment, which was 3 months after surgery, the regenerated lens was almost the same in size as the intact lens, with relatively normal morphology (Figure 1R,U).

**Figure 3:**
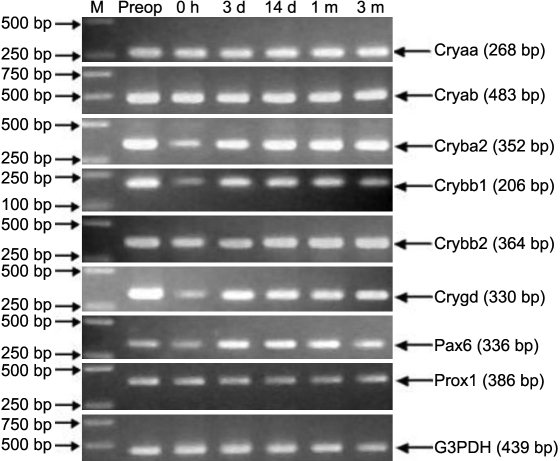
RT–PCR analysis for crystallin genes of *Cryaa*, *Cryab*, *Crybb1*, *Crybb2*, *Cryba2*, and *Crygd* (encoding αA-, αB-, βB1-, βB2-, βA2-, and γD-crystallin), and the transcription factors *Pax6* and *Prox1*. The lens tissue is dissected at pre-operation, 0 h, 3 days, 7 days, 14 days, 1 month, and 3 months after ECLE and is then assayed. The size of specific PCR products is shown in parentheses.

**Figure 4:**
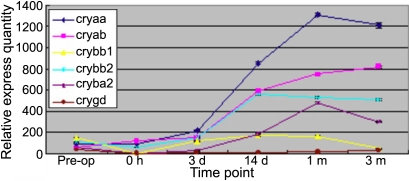
Real-time PCR analysis mRNA of *Cryaa*, *Cryab*, *Crybb1*, *Crybb2*, *Cryba2,* and *Crygd*. The lens tissue is dissected at pre-operation, 0 h, 3 days, 7 days, 14 days, 1 month, and 3 months after ECLE and is then assayed.

To survey the expression of crystallin in lens regeneration, we detected the mRNA of *Cryaa*, *Cryab*, *Crybb1*, *Crybb2*, *Cryba2*, and *Crygd* by RT–PCR analysis and real-time PCR. At any time point, the mRNA of those crystallin could be detected by RT–PCR (Figure 3) and confirmed by sequencing the PCR products (data not shown). Crystallin accumulated progressively and its relative expression peaked at 1 month after ECLE (Figure 4).

### Transcription factors

**Figure 5:**
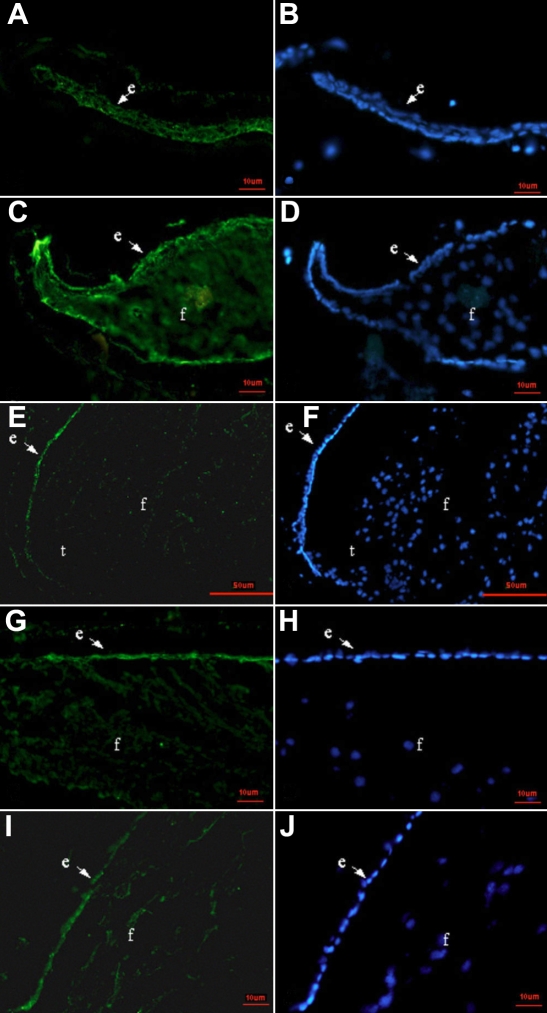
Pax6 immunolocalization of rat lens regeneration from 3 days to 3 months after ECLE. Immunostaining for the Pax6 protein (green) with nuclear counterstain DAPI (blue). Pax6 protein, normally found in epithelial cells and new lens fibers, is similar in lens regeneration and normal lens development. The lens tissue is stained for Pax6. Note that the anterior capsule adheres to the posterior capsule 3 days after ECLE (**A**, **B**); Pax6 is expressed in the lens epithelium and differentiating fibers on day 7(**C**, **D**); Pax6 is predominately located in the lens epithelium, and there is a low level of expression in the newly differentiated lens fiber cells on day 14 (**E**, **F**; **G**, **H**);and finally, the level of Pax6 expression is reduced in the denucleated fibers 1 month after ECLE (in the lower right corner of **I**, **J**). Abbreviations: e, lens epithelium; f, lens fiber cells; t, transition zone.

**Figure 6:**
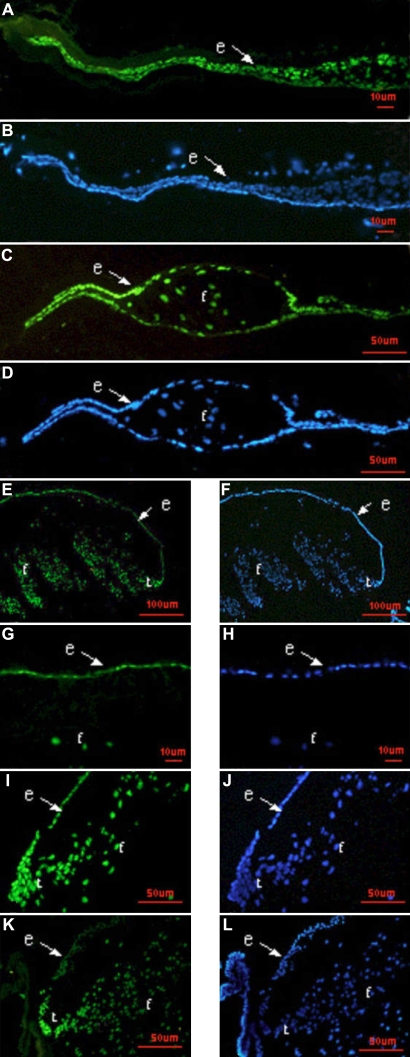
Distribution of Prox1 protein during the development of lens regeneration in a rat model. Immunostaining for the Prox1 protein (green) with nuclear counterstain DAPI (blue). Prox1 protein, normally found in epithelial cells and lens fibers, appears similar in lens regeneration and normal lens development. Note that the anterior capsule adheres to the posterior capsule on day 3 (**A**, **B**), the fiber differentiation is obvious on day 7 (**C**, **D**), and the Prox1 is found in the LECs and differentiating lens fibers on day 14 (**E**, **F**). One month after ECLE, Prox1 is mainly located in the nucleus of LECs and to a lesser extent the cytoplasm of LECs in the region of anterior capsule (**G**, **H**; I, **J**). While in the germinative zone, Prox1 is absolutely located in the nucleus of LECs, in the lens fiber cells, Prox1 is strictly located in the nucleus. Three months after ECLE, Prox1 is mainly expressed in the transition zone (**K**, **L**). Abbreviations: e, lens epithelium; f, lens fiber cells; t, transition zone.

**Figure 7:**
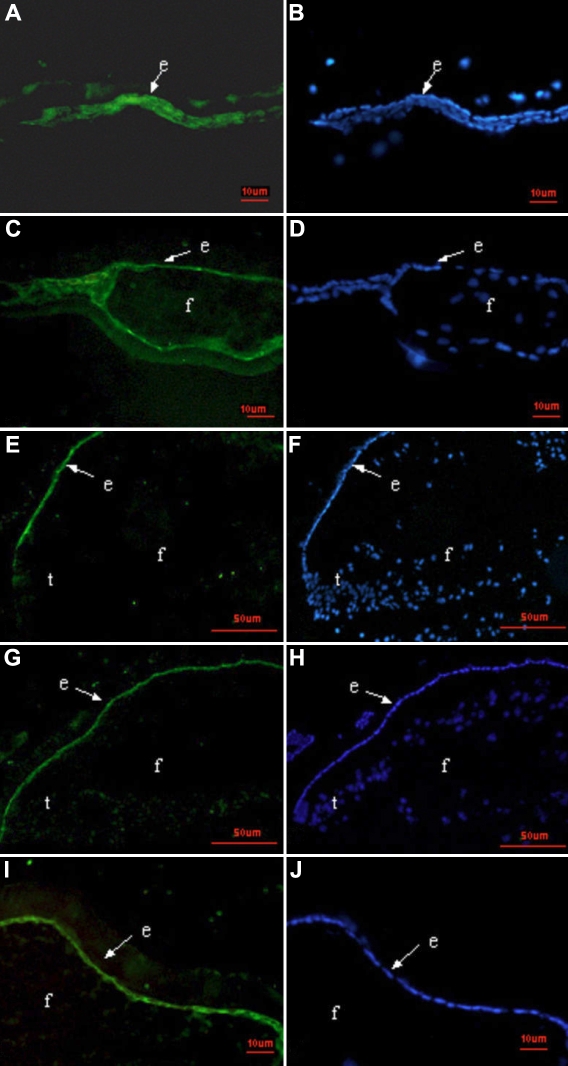
The location of Foxe3 during the development of PCO in rat. Immunostaining for the Foxe3 protein (green) with nuclear counterstain DAPI (blue). Foxe3 protein, normally confined to the anterior epithelial cells, is the same as in lens regeneration and as it is in normal lens development. Note that the anterior capsule adheres to the posterior capsule on day 3 (**A**, **B**), no Foxe3 positive stain is found in the differentiating fibers at day7(**C, D**), and Foxe3 is strictly located in the lens epithelium on day14 (**E**, **F**) and 1 month after ECLE (**G**, **H**; **I**, **J**). Abbreviations: e, lens epithelium; f, lens fiber cells; t, transition zone.

Three DNA binding proteins, Pax6, Prox1, and Foxe3, were detected at all time points by immunofluorescence. The expression patterns of these three proteins in lens regeneration were similar to those shown in normal lens development. Pax6 protein, which was detected in the cytoplasm and nucleus, was predominately expressed in the LECs and to a lesser extent in the lens fiber cells (Figure 5). Pax6 protein was also expressed in the cornea, iris, and retina (data not shown). Prox1 protein was localized at an increased level in the LECs and regenerated lens fibers and was concentrated in the nuclei of newly differentiating lens fiber cells and LECs in the germinative area (Figure 6). In the LECs of the anterior capsule, there was weak expression of Prox1 in the cytoplasm and dense expression in the nuclei (Figure 6G,H), while in the lens fiber cells, Prox1 protein was strictly expressed in nuclei. Prox1 protein was also expressed in the inner nuclear layer of the retina (data not shown). Foxe3 protein is mostly located in the cytoplasm of LECs during lens regeneration (Figure 7).

**Figure 8:**
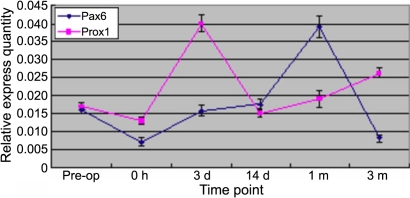
Real-time PCR analysis mRNA of *Pax6* and *Prox1*. The lens tissue is dissected at pre-operation, 0 h, 3 days, 7 days, 14 days, 1 month, and 3 months after ECLE and is assayed.

mRNA expression patterns of *Pax6* and *Prox1* by RT–PCR are shown in Figure 3, and PCR products were also confirmed by sequencing analysis (data not shown). The relative expression quantities of *Pax6* and *Prox1* immediately after ECLE decreased noticeably as visualized by real-time PCR, shown in Figure 8. Three days after surgery, the mRNA expression of *Prox1* increased significantly. Fourteen days after ECLE, the mRNA expression of *Prox1* was less than that at 3 days after ECLE. From then on, the relative expression quantity of *Prox1* increased slowly until 3 months after ECLE. mRNA expression of *Pax6* increased gradually following ECLE, peaking at 1 month and decreasing obviously at 3 months.

As the exact mRNA sequence of rat *Foxe3* was unavailable from the current Genebank database, we failed to detect the expression of *Foxe3* by PCR, though several different pairs of primers were tried.

## Discussion

### Cell differentiation and lens growth are the mechanisms of lens regeneration

In this study, as previously reported by other investigators [1-3], lens fiber differentiation was found to follow a process similar to embryological development with proliferation of epithelial cells along the anterior and posterior capsule, elongation of the posterior epithelial cells, and differentiation of epithelial cells into lens fibers. Once new lens fibers are formed, they are gradually compacted as newer fibers are produced and the central mass of the lens builds. we observed that LECs were left behind under the anterior capsule immediately after ECLE. Lens fiber differentiation had occurred in the peripheral capsular bag in all rats 3 days after ECLE. The nuclei of LECs lining the posterior capsule migrated away from the basement membrane at 7 days after ECLE. One month after surgery, all capsular bags were filled with new semitransparent lenticular structures, displaying an established equator with well differentiated bow regions.

In this study, we found that the regenerated lenses were irregular in shape and translucent due to lack of lens growth at the site of the anterior capsulotomy and its adhesion to the posterior capsule. The arrangement of lens fibers and thus the transparency of the regenerate was improved in the present study when the continuous circular capsulorhexis of the anterior capsule was smaller and more regular. Several factors were shown to affect lens regeneration, such as age of the animal, inflammation, and the size and the shape of the capsulotomy [8].

Extracapsular lens extraction is a not only a surgery but also a stress-inducing situation for the animal. Epithelial to mesenchymal transition (EMT) has been observed in mice undergoing the initial stages of regeneration, indicating that the process entails an initial phase of repair and of lens differentiation [3]. Microarray analysis revealed that initially there is a response to injury, extensive matrix remodeling, and severe downregulation of genes encoding lens structural proteins. The patterns gradually returned to normal three weeks after surgery [9].

### Protein composition of regenerated lenses

During lens development, the morphological transition between epithelial cells and fiber cells coincides with specific, regulated changes in crystallin gene expression. Crystallin constitutes about 90% of soluble lens proteins. All mammals examined to date have three major crystallin gene families: α-, β-,  and γ-crystallin, each of which includes several members [10]. Of these families, α-crystallin, produced by epithelial and fiber cells, is first expressed at the lens vesicle stage; β- and γ-crystallin are unique to fiber cells, and β-crystallin is an early marker of fiber cell differentiation which begins on embryonic day 11 (E11.0) in mice, with γ-crystallin generally being expressed one or two days later. Previous studies showed evidence of fiber differentiation in rabbit and mouse models via stains that used a lens fiber-specific β-crystallin antibody [3]. To survey the expression of crystallin in lens regeneration, we detected the mRNA of *Cryaa*, *Cryab*, *Crybb1*, *Crybb2*, *Cryba2*, and *Crygd* by real-time PCR and found progressive accumulation of crystallin after ECLE. This is a hallmark of lens fiber cell growth and maturation. To confirm the identities of the crystallins found, we sequenced every crystallin PCR product and confirmed its identity by comparison to the sequence of objective genes in the gene bank (data not shown).

Staining regenerated lenses with a crystallin antibody would help compare their morphology and arrangement; however, fluorescent immunohistochemical labeling of the adult vertebrate lens has been problematic due to difficulties in preparing high quality 6–8 mm sections because of the physical properties of the lens. These problems include poor fixative penetration in unsectioned tissue, difficulty preparing sections with an acceptable morphology, non-specific binding of antibodies due to high protein concentration within the lens, light scatter and autofluorescence induced by lens proteins [11]. On the other hand, there are more than ten members of crystallin protein families expressed in the mammalian lens. For these reasons, we did not detect crystallins with immunostaining in this study. Further work on crystallin research in regenerated lenses will provide more insights into this process.

### Transcription factor expression in regenerated lenses

The lens has provided a relatively simple structure for the study of developmental mechanisms. Molecular genetics techniques have been applied to the analysis of lens induction, which has advanced our understanding of lens formation. A series of transcription factors involved in early lens development have been identified. These include Pax6, Prox1, Foxe3, Mafs, Sox2, and others. These transcription factors are critical for lens morphogenesis and lens fiber differentiation.

Pax6 is one of the major regulators of the crystallin genes and other transcription factors. Its gene dosage and appropriate levels of transcriptional activity have been shown to be important in ocular development [12,13]. During lens development, Pax6 regulates the expression of various crystallins and is required for lens fiber differentiation. During newt lens regeneration, Pax6 plays a similar role in regulating crystallin expression, and it regulates proliferation but not differentiation at later stages of regeneration [14]. In this study, there was significant regulation of *Pax6* during rat lens regeneration. *Pax6* mRNA was highly expressed at 1 month after lens extraction, which correlated with crystallin expression. At 3 months after lens extraction, the expression of *Pax6* decreased noticeably. Pax6 protein was detected with immunofluorescence and found primarily in the epithelium. It was also detectable in newly differentiated lens fibers at levels that decreased as fiber differentiation proceeded. As we know, the loss of *Pax6* expression in lens fiber cells is essential for normal fiber cell differentiation and crystallin gene expression [5]. The role of *Pax6* in mammal lens regeneration is an area for further research.

Prox1, a divergent homeodomain protein, is expressed initially in the early lens placode and is upregulated during fiber cell differentiation [15,16]. Generation of the *Prox1* null allele in mice reveals that this transcription factor is essential for the differentiation of lens fiber cells. Prox1protein shows upregulation in the dorsal iris during the process of newt lens regeneration [17]. During rat lens regeneration, *Prox1* mRNA was upregulated during the very early stages of regeneration at day 3. Day 3 marks an early response to lens removal and is expected to be characterized by LECs proliferation and early stages of fiber cell differentiation (Figure 2C-E). At that time, more *Prox1* mRNA may be needed for initiation or promotion of fiber cell differentiation. Prox1 protein is found in both the nucleus and to a much lesser extent the cytoplasm of LECs, and it is confined to the nucleus of LECs in germinative areas and regenerated lens fibers. The role of Prox1 in mammal lens regeneration is another area in which further research is warranted.

*Foxe3* is expressed in the early stages of lens induction, turns off its expression in differentiating fiber cells, and remains active only in the proliferative and relatively undifferentiated cells of the anterior lens epithelium. Previous studies showed that all three crystallin families were activated in a dysgenetic lens with defective Foxe3, demonstrating that Foxe3 was not necessary for their expression [18]; however, Foxe3 is essential for promoting survival and proliferation of lens epithelium cells [19]. The most common late complication following cataract surgery is posterior capsule opacification (PCO). Since the cause of PCO is overgrowth of LECs onto the posterior face of the lens capsule, PCO would be repressed if the Foxe3 gene is silenced by molecular biology technology. Foxe3 is an ideal target gene for gene therapy in treating PCO because its expression is limited to LECs.

With this approach, we demonstrated the utility of a rat model in the study of mammalian lens regeneration at the molecular level. Regenerated lenses contain proteins and transcription factors similar to those in normal lenses. Similar events might take place during development and regeneration of the lens, but inductive interactions seen during lens development are not necessary for lens regeneration. The goal of this study was to identify transcription factors and crystallins that might be expressed during mammal lens regeneration. Extension of this study will lead to the establishment of databases and will help to elucidate the mechanisms responsible for inducing regeneration and PCO at the genetic level.
